# Cell Proliferation in the Presence of Telomerase

**DOI:** 10.1371/journal.pone.0004622

**Published:** 2009-02-27

**Authors:** Krastan B. Blagoev

**Affiliations:** National Science Foundation, Arlington, Virginia, United States of America; Center for Genomic Regulation, Spain

## Abstract

**Background:**

Telomerase, which is active early in development and later in stem and germline cells, is also active in the majority of human cancers. One of the known functions of telomerase is to extend the ends of linear chromosomes, countering their gradual shortening at each cell division due to the end replication problem and postreplication processing. Telomerase concentration levels vary between different cell types as well as between different tumors. In addition variable telomerase concentrations will exist in different cells in the same tumor when telomerase inhibitors are used, because of limitations of drug delivery in tissue. Telomerase extends short telomeres more frequently than long telomeres and the relation between the extension frequency and the telomere length is nonlinear.

**Methodolgy/Principal Findings:**

Here, the biological data of the nonlinear telomerase-telomere dynamics is incorporated in a mathematical theory to relate the proliferative potential of a cell to the telomerase concentration in that cell. The main result of the paper is that the proliferative capacity of a cell grows exponentially with the telomerase concentration.

**Conclusions/Significance:**

The theory presented here suggests that long term telomerase inhibition in every cancer progenitor or cancer stem cell is needed for successful telomere targeted cancer treatment. This theory also can be used to plan and asses the results of clinical trials targeting telomerase.

## Introduction

Telomeres protect the ends of linear chromosomes from being recognized by the DNA repair system as double strand breaks in need of repair[1], [2], [3]. In the absence of a lengthening mechanism, during DNA replication telomeres lose nucleotides partly due to the inability of DNA polymerase to replicate their ends[4], [5] and partly due to post-replication processing needed to create a single strand overhang[6], which is part of the telomere protective structure known as shelterin[7]. In the absence of a telomere extension mechanism, a dividing cell will acquire a short telomere incapable of maintaining the shelterin integrity. This may trigger a p53 dependent checkpoint response leading to cell cycle arrest[8], [9], [10], [11]. Cells, however, have developed a mechanism for countering this gradual loss of telomeric DNA. In some organisms telomere recombination has emerged as a telomere maintenance mechanism[12], while in others, including humans, telomere length homeostasis is accomplished by telomerase, a ribonucleoprotein complex that provides RNA template sequence for telomeric DNA extension[2], [13]. Normal human somatic cells have telomerase levels below the level required for telomere maintenance and their telomeres shorten with each cell division[14]. There is substantial evidence that short telomeres limit cell's ability to proliferate and that gradual telomere shortening in normal somatic cells leads to their finite proliferative capacity[8], [15]. Cancer cells on the other hand acquire infinite or very large proliferative potential (PP) (the potential number of cell divisions a cell can undergo before entering senescence) by reactivating a program for telomere homeostasis[16]. Telomerase is also detectible in stem cells[17], and these cells have large, but limited proliferative capacity. In most tumours, cancer cells re-express telomerase. In some cancers, there is no detectible telomerase and these cancer cells use an alternative lengthening of telomeres (ALT), mechanism for telomere maintenance. ALT is believed to be recombination based[18], [19], [20], [21] and is characterized by long and heterogeneous telomeres ranging from 2 kb to 50 kb[22], extra-chromosomal telomere repeats[23], and ALT associated promyelocytic leukimia (PML) nuclear bodies that contain PML protein, TRF1, TRF2, replication factor A, Rad51, and Rad52[24]. There are also cancer cells that use neither telomerase, nor have the characteristic signatures of ALT and in these instances it is not clear how telomeres are replenished. There is some evidence that both telomerase and ALT might be active in different cells of the same tumor[25]. Because telomerase [6] is expressed in most human cancers, it is an attractive therapeutic target[26], [27], [28], [29]. Telomerase inhibition does not typically reactivate the ALT mechanism, although in one instance an ALT phenotype emerged after telomerase suppression[11]. In addition suppressing simultaneously mTerc and Wrn in mouse cells leads to increased telomere-telomere recombination rates and an activation of ALT[30]. Telomerase re-activation seems to inhibit the recombination based maintenance mechanism in human cells[31].

At each cell division telomere length regulation consists of basal telomere loss and telomerase facilitated telomere gain. In short this can be expressed as
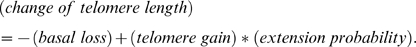



The extension probability in *Saccharomyces cerevisiae* [32], [33], [34], human cancer cells, and in telomerase positive, normal human fibroblasts[32] has been quantified recently. The data suggests that the extension probability or the extension frequency is a sigmoid type of curve and was well fitted by a logistic regression. In wild type cells with sufficient telomerase expression for maintaining telomere homeostasis, telomeres are maintained at an equilibrium length. In *S. cerevisiae* this equilibrium length is approximately 300 base pairs (bp)[33], while in immortalized human cells it is between 5000 and 15000 bp[14]. The basal telomere loss in *S. cerevisiae* is 3 nucleotides (nt) per generation[35] and while in human cells it is between 50 and 200 bp[14]. Larger telomere rapid deletions (T-RD) may occur as well, due to DNA double strand breaks or errors during DNA replication[36]. The number of telomere repeats added by telomerase in a single cell cycle *in vivo* varies from few to more than a hundred nucleotides in *S. cerevisiae* [33] and up to 800 in human super-telomerase cells[37]. Telomerase adds nucleotides to *S. cerevisiae* telomeres in late S phase, but does not replenish all telomeres at each cell replication either because it might not be available at all telomeres during that time or because when available at a telomere it may not be able to extend the telomere. Recent data suggests that in budding yeast[33] telomeres switch back and forth between two states: extendible or open state, which allows telomerase to associate with the telomere and a nonextendible or closed state, which prevents telomerase from associating with the telomere. This binary response suggested in this study is consistent with the sigmoid function used to fit the data. The oscillation frequency between these two states is higher for shorter telomeres and this leads to a higher probability for a telomerase complex to associate with these telomeres. Even when telomerase associates with a telomere it might not extend that telomere. Whether or not a telomerase associated with a telomere processes it or not depends on its length[32], [33], [37] and perhaps on the state of the shelterin complex. In *S. cerevisiae* the repeat addition processivity (the number of telomere repeats added per round of DNA replication) is higher at shorter telomeres[32], but is lower than in human cells. In human cells telomerase concentrations correlate with increased repeat addition processivity[38]. In cells in which telomerase is partly inhibited a new equilibrium length is established by a feedback control mechanism as shown in *S. cerevisiae* [33]. Recently telomerase in human cells was expressed beyond the physiological limit [37] and in these super-telomerase cells the telomere extension dynamics did not seem to slow down, continuing with the same average rate for more than 60 population doublings [37]. This constant rate of elongation suggests that in these cells the combined probability for a telomere to be in an open state times the number of base pairs added to the telomere during an elongation event is a constant that is larger than the basal telomere loss. In the HEK-293 human cancer cell line the number of telomerase complexes, 50[39], is approximately few times smaller than the number of telomeres, suggesting that the telomerase concentrations are limiting[40], [41].

## Results

**Figure 1:**
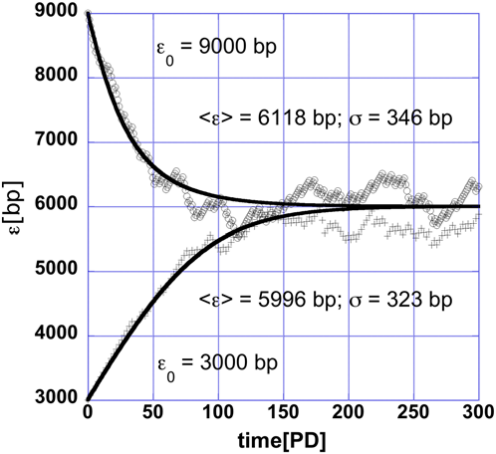
In this figure the time dependence (in cell divisions) of the average telomere length ε is shown. Because the initial length of the two representative telomeres shown in the figure is longer (shorter) than the homeostatic length the telomeres gradually approach the homeostatic length. Adaptive control at the telomere guarantees that short telomeres are extended and long telomeres are shortened to the average homeostatic length. The initial telomere length, the average steady state telomere length and the square root of the variance are shown for two telomeres in the presence of telomerase. While at the steady state, the square root of the variance is small compared to the average telomere length. As the telomere shortens below 1000 bp these two quantities will become comparable and large fluctuations may produce a sub-critically short telomere and trigger p53 independent checkpoint response. The continuous model is shown with continuous lines.

**Figure 2:**
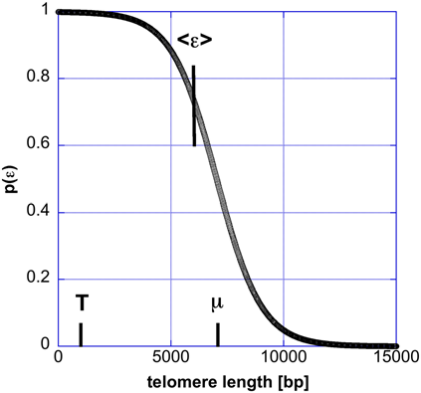
The feedback control function, maintaining the telomere length, is shown. On this figure the dependence of the probability (or the frequency), *p*, for occurrence of the open state on the telomere length ε (in base pairs) is shown. The parameter μ in this function is the telomere length at which the probability for the extending state is ½. The parameter T = 1000 determines the slope of the sigmoid. The choice of T is close to the maximum possible value for telomere homeostasis at telomere length of 6000 bp (see the text for an explanation).

In Fig. 1 the probabilistic and deterministic length control dynamics is shown for two telomeres with different initial lengths: one shorter and one longer than the steady state length. The feedback control steadily increases the length of the shorter and decreases the length of the longer telomere. The speed of telomere elongation or depletion is larger the farther a telomere is from the steady state and becomes zero for telomeres with the steady state length. For fixed steady state length this speed is controlled by the parameter T in Eqn.(4). The speed at which the telomeres approach the steady length depends on the parameters T and μ in the logistic response (Fig. 2). For small T, e.g. = 1, μ = 6001 and the speed approaches the maximum speed determined by the basal loss (100 bp/cell divison this model). This is because the logistic function is zero (one) for longer (shorter) than the steady length telomeres most of the time. Therefore the curves in Fig. 1, represent the slowest telomere length dynamics. The choice of parameters in the logistic probability function is consistent with current data for *S. cerevisiae* [*33*], but for human the telomere length is not known.

**Figure 3:**
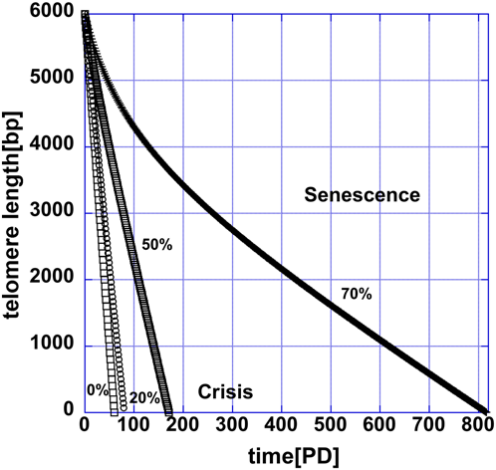
In the presence of telomerase inhibitor, telomerase is incapable of preventing the appearance of a critically short telomere. The attrition of telomeres at different telomerase inhibition level is shown for several telomeres with the same initial length. The time to the crisis point for each cell determines the proliferative potential of that cell.

**Figure 4:**
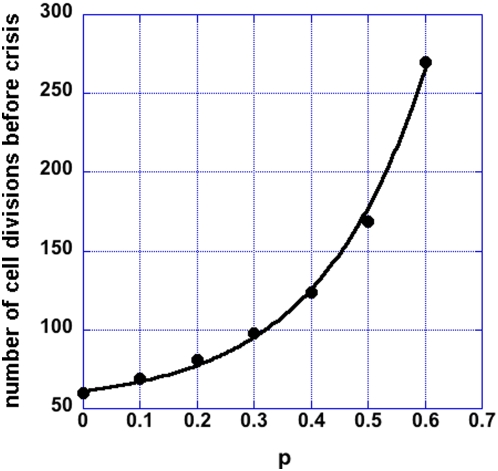
The average proliferative potential or the number of cell divisions before crisis of a population of cells is shown as a function of the degree of telomerase inhibition *p*. The parameter *p* describes the availability of telomerase and varies between 0 (no telomerase is available) and 1 (all telomerase is available). The proliferative potential is averaged over 1000 cells for each inhibition level and the functional dependence is well approximated by an exponential function in the interval p = 0.0 to p = 0.6: V = V_0_+V_1_exp(p/p_0_) (V_0_ = 52, V_1_ = 8, and p_0_ = 0.17, R = 0.99816).

Lowering the telomerase concentration leads to faster decrease in telomere length (the fastest decrease is set by the basal loss rate). The first telomere reaching a critical length is expected to trigger a p53-dependent checkpoint cellular response. In human cells this critical length is 2000–4000 bp. However, in most cancers the p53 pathway is inactivated and the telomeres continue to decrease in length until the crisis point is reached. At the crisis point the short telomeres are only few hundred base pairs in length[42] and therefore telomerase inhibition is more effective in cancers with an intact p53 response. In Fig. 3, the telomere erosion is shown for telomeres with initial length of 6000 bp, corresponding to cells with different degree of telomerase suppression. In this figure it is assumed that when the telomerase-telomere fraction is 50/92 the telomeres are maintained at their steady state length of 6000 bp. The critical telomerase concentration below which telomeres reach zero length is 73.6% of 50/92. The time that it takes to reach crisis (0 in this figure) and thus the PP decreases with decreasing telomerase concentration. The relation between the time to crisis and the telomerase concentration is shown in Fig. 4. Here the normalized telomerase concentration p is relative to the telomerase-telomere fraction π_0_ = 50/92 at which the PP is infinite, i.e. π = (50/92)p. The dependence of the PP is well approximated by an exponential in this parameter range: 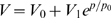, with V_0_ = 52, V_1_ = 8, and p_0_ = 0.17. For p>0.736, the PP of a cell is infinite, i.e. the telomeres are maintained at a nonzero length.

Telomere elongation by telomerase or for that matter by any other telomere maintenance mechanism must be such that no critical or subcritical telomeres occur during this process. This means that the number of base pairs added per elongation event minus the telomeric basal loss must be small compared to the telomere size. On the other hand if this number is too small, the recovery of a short telomere to the steady state length would be too slow and a few recombination events may bring the length of a telomere below the critical length triggering a checkpoint response. If, however, this number is too large, the frequency at which the extending state occurs needs to be low. Again, these large fluctuations may lead a short telomere to the critical length, because of the high probability that a telomere may not be extended during several consecutive cell divisions. The parameters that govern the telomere length homeostasis, the basal loss, the telomerase concentration, the number of nucleotides added by telomerase, the equilibrium telomere size, and the probability for an extendable state have been selected in a range that guarantees robust telomere maintenance dynamics despite the naturally occurring noise fluctuations and this may be the clue to why telomerase levels are tightly regulated.

In the HEK-293 human cancer cell line, 50 telomerase complexes are present[39] and the availability of telomerase at a particular telomere depends on whether or not other telomeres are being extended. The physical mechanism by which telomerase is being directed preferentially to short telomeres is not clear, but some details are emerging[43]. In *S. cerevisiae*, because of the short telomeres (few hundred base pairs), a telomerase-telomere counting mechanism based on the number of nucleotides or of the number of telomere bound proteins seems to exist. In human cells telomerase maintains the telomeres at several thousand bp and the counting mechanism, if it exists, remains unknown. One possibility is that the shorter the telomere the earlier it becomes available for elongation in S phase[44] and telomerase binds to it. The telomerase will bind in a successive order from short to long telomeres. This may explain why short telomeres are elongated first and why telomerase is maintained at lower numbers than the number of chromosome ends. However, the time of telomere replication is not correlated with their length[45] and a simple relation between replication time and telomere extendibility is not likely to exist.

In *S. cerevisiae*, the nonlinear adaptive control that telomeres perform through a feedback loop on their extension rate is performed by the frequency at which an extendible state occurs in other organisms the mechanism is currently unknown. Because, the steady state telomere length in human cells is much longer than in *S. cerevesiae* the length control in this case might be accomplished by the adaptive extension length of the added telomere repeats by telomerase and not by the frequency of occurrence of the extendible state. This, alternative mechanism is also captured by the model introduced here, because 
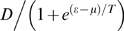
 in Eqn. (4) can be interpreted as the length of the added telomere repeats by the telomerase complex. This means that the model is more general than the specific mechanism of telomere length homeostasis. This paper is concerned with the dynamics of telomere attrition caused by telomerase depletion. In this case the speed at which telomerase replenishes a telomere is related to the probability with which the extending state occurs and on the probability of a telomerase complex being at the telomere. Below a critical telomerase concentration the telomere cannot be sufficiently replenished and will become eventually critically short, despite the increasing frequency, with which it is in an extendible state. Lower telomerase concentration will lead to less frequent extension events and consequently to slower extension.

For cells with abundance of telomerase the telomeres are extended beyond that predicted by the theory steady state length[37]. It is possible that the telomere length control system has an upper limit on counting telomere length above which it is incapable of tracking the length of a telomere and therefore the probability for an open state above this limit is a nonzero constant[37] explaining the results in human super-telomerase cells. This effect can be incorporated in the theory, but more studies are needed to understand how does the extension rate in super-telomerase cells, relates to their telomerase concentration. Currently available data is inconclusive regarding the relation between the telomerase concentration and the rate of telomere extension.

One of the conclusions of this paper is that in clinical settings, telomerase inhibitors need to be administered robustly for a long time: more than 250 cell divisions, if telomerase is suppressed only 60%. However, low levels of the RNA component of telomerase lead to a deleterious condition, dyskeratosis congenital, characterized by short telomeres and premature death from bone marrow failure[46]. A question that can be addressed by the model presented here is the optimal drug administration schedule that could limit the effect of telomerase inhibition on the replicative capacity of the hematopoietic cells and maximize the suppression of the tumour proliferation.

In clinical settings the grade of a tumour is often quantified histologically in terms of the mitotic index[47], [48], i.e. the fraction of mitotic spreads visible under a microscope in a given slice of tissue. Most cells in real tumours do not divide at all times, because otherwise a single cell after 60 divisions will create approximately 10^20^ cells, more than the number of cells in the human body. How is the telomere derived proliferative potential related to the mitotic index? The mitotic index of a cell population is between the smallest and the largest proliferative potential. The lowest proliferative potential of a dividing cell after *n* cell divisions is log_2_(*n*+1), when only one of the two daughter cells divides at each cell division. This will correspond to a stem cell renewing itself and a non-dividing progenitor cell. The maximum is *n*, when every daughter cell divides at each cell division. The mitotic index depends on many factors that limit and stimulate cell proliferation and telomere length is one of them. In a tumour in the presence of telomerase inhibition different proliferating cells will most likely be exposed to a different level of telomerase inhibition and will require different time to stop dividing. Several studies have shown positive correlations between the median survival time of cancer patients and the average telomere length of the tumour cells[49], [50]. Telomerase expression levels are also negatively correlated with the median survival time[51]. Therefore, the telomere related proliferation potential is positively correlated with the mitotic index and it will be interesting to know how the dynamics of the mitotic index is related to the telomerase inhibitor concentration and duration. This will allow the quantification of the relation between the tumour malignancy and the proliferative capacity in cancer.

## Materials and Methods

A minimal model capturing the biological observations is described by the equation 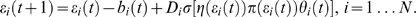
(1)


Here N is the number of telomeres, ε_i_(t) is the length of the *i*th telomere at time t, b_i_(t) is the telomere basal loss, η(ε_i_(t)) is the probability that a telomere is in an extendible state. D_i_ is the number of base pairs added to the *i*th telomere during an elongation event, π is the probability that telomerase is in the neighbourhood of the *i*th telomere, and θ_i_ is the probability for telomerase association with the *i*th telomere. The binary function σ is 1 with probability η(ε_i_(t))π(ε_i_(t))θ_i_(t) and 0 with probability 1−η(ε_i_(t))π(ε_i_(t))θ_i_(t), because the three probabilities are independent.

Two regimes of telomerase-telomere interaction can be identified that depend on the telomerase concentration. At a very high telomerase concentration, as in the supertelomerase cells, multiple telomerase complexes can be available for the elongation of each telomere and every time an extendible state occurs telomerase is available to associate with each telomere. The telomere length in this regime can be limited by the frequency at which the extendible state occurs and by the telomerase repeat addition processivity. The extension of different chromosome ends is uncorrelated, because the number of telomerase complexes is larger than the number of telomeres. As the number of telomerase complexes is reduced below the number of short telomeres, a new regime of adaptive control occurs in which telomerase levels limit the telomere length. In this situation the telomere length is controlled by the probability for a telomerase complex to be at a particular telomere, by the length dependent probability of this telomere to be in an open state at that time, and by the telomerase processivity. In *n* number of cell divisions the telomere will be extended to a length 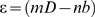 where *m* is the number of times the telomere was in an extending state, *D* is the number of added base pairs (bp), and *b* is the number of bp lost at each cell division (basal telomere loss). In the model used here for N equal telomeres and M telomerase molecules, the probability π(ε_i_(t)) is assumed to be equal to 1 if telomerase is over expressed (as in super-telomerase cells) i.e. M≥N and it is equal to M/N if M<N. This simple approximation is based on the assumption that telomerase is reliably “delivered” to the telomeres. The probability that a telomere is in an open state, η(ε_i_(t)) in *S. cerevisiae*, is well approximated by a logistic regression[33], which can be viewed as describing the statistical properties of a system with binary occupation of its states, 0 or 1 (nonextendible, extendible). The logistic function specifies the type of feedback control that the telomere length exercises on its elongation and in budding yeast for fixed telomerase concentration
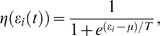
(2)where μ, and T are constants (Fig. 2). When the telomere length is equal to μ, the probability for an open state is equal to the probability for a closed state = ½, while T controls the statistical fluctuations. The parameters in the model are fixed by average experimental values: average telomere length −6000 bp (appropriate for human cancer cells); average basal loss −100 bp; number of telomerase complexes at equilibrium −50; number of base pairs added by telomerase −250. The average extension rate per cell division can be used to determine the parameters in the feedback control function. Assuming that there are 50 telomerase complexes and 92 telomeres in a cell, the steady state is determined by the condition that on the average the basal loss balances the added telomere base pairs by telomerase[33]. Mathematically this means that
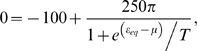
(3)where the factor π accounts for the ratio of the number of telomeres to the number of telomerase complexes and ε_eq_ = 6000 is the telomere equilibrium length. In this approximation it is assumed that all telomeres are of equal length and ε_i_ is replaced by ε. From this equation, it follows that the parameters in the feedback control function can be fixed to: μ = 7025 bp (corresponding to a probability for an open state = ½) and T = 1000 bp for π = 50/92. Here π is equivalent to the telomerase concentration expressed as a fraction of the number of telomeres. Telomerase inhibition results in lower values of the steady state telomere length, ε_eq_.

The theory discussed in the previous section (Eqn. (1)) can be cast in the form of a differential equation when the time scale of interest is large compared to the cell division time. In this limit the model is
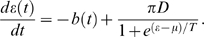
(4)


This differential equation cannot be solved explicitly, but several limits on the parameters of the logistic function can be set from it. When the telomere length is equal to the steady state length (ε_eq_ = 6000), the left hand side of Eqn. (4) is zero. This leads to the following relation between the telomerase concentration, π and the parameters μ and T:
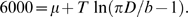
(5)


At π = 50/92, b = 100 bp/cell division, and D = 250 bp/cell division the relation between μ and T is μ = 6000+1.025T. On the other hand Eqn. (5) sets a limit on the parameter T because π is a concentration with a maximum value of 1 when ε->0. The relation is π = 0.4+e^−μ/T^. This means that 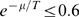 or equivalently T≤μ/5.12 = 6000+1.025T. From the last relation follows that the maximum value of T and μ, which will guarantee that the steady state length of the telomere is 6000 bp are T_max_ = 1467 bp and μ_max_ = 7504 bp respectively. The parameter T sets the slope of the sigmoid curve displayed in Fig. 2 and μ sets the value at which the probability for an open state is equal to ½. This result means that to maintain telomere length at 6000 the slope of the curve describing the frequency extendible state cannot be larger than a certain number. This slope is given by the derivative of the π with respect to ε at ε = μ and is equal to 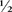. In this study the value T = 1000 is assumed, which is close to the maximum value and the results presented here are a lower limit to the speed with which telomerase extends a telomere, i.e. the worst case scenario for treatment and more quantitative data with different human cells will be useful to better quantify the values of the parameter in the theory.
